# An evaluation of progressive amyloidogenic and pro-inflammatory change in the primary visual cortex and retina in Alzheimer's disease (AD)

**DOI:** 10.3389/fnins.2014.00347

**Published:** 2014-11-12

**Authors:** James M. Hill, Prerna Dua, Christian Clement, Walter J. Lukiw

**Affiliations:** ^1^Louisiana State University Neuroscience Center and Departments of Ophthalmology and Pharmacology, Louisiana State University Health Science CenterNew Orleans, LA, USA; ^2^Department of Health Information Management, Louisiana State UniversityRuston, LA, USA; ^3^Department of Natural Sciences, Infectious Diseases, Experimental Therapeutics and Human Toxicology Lab, Southern University at New OrleansNew Orleans, LA, USA; ^4^Department of Neurology, Louisiana State University Health Science CenterNew Orleans, LA, USA

**Keywords:** Aβ42 peptides, Alzheimer's disease (AD), amyloid, inflammatory signaling, retina, spreading, visual system

## Overview

Sporadic Alzheimer's disease (AD; idiopathic, of unknown origin) is associated with dysfunctional gene expression in the limbic system and entorhinal cortex of the brain that drives amyloidogenesis, pro-inflammatory signaling, alterations in innate-immunity and related AD-type neuropathology (Colangelo et al., 2002; Lukiw, 2004; Ginsberg et al., 2012; Kikuchi et al., 2013). While the primary visual cortex (Brodmann Area 17) and the retina appear to be initially spared of AD-type changes that devastate the hippocampal CA1 and temporal lobe neocortex (Brodmann Area 22), these primary sensory and signal processing elements of the visual system become more involved as AD progresses. Recent data indicate that in moderate to late-stage AD, pro-inflammatory and amyloidogenic pathology spreads along the entorhinal-primary visual sensory cortex-thalamic-retinal axis, and this may be responsible in part for the complex visual disturbances, such as spatial and visual agnosia, facial identification problems, perceptual disturbances, and visual hallucinations, associated with end-stages of the AD process (Cui et al., 2007; Dehabadi et al., 2014; Tzekov and Mullan, 2014; Zhao et al., 2014). This “Opinion paper” will comment on current trends in our understanding of specific amyloidogenic and pro-inflammatory changes in the human primary visual cortex and retina as AD advances with particular reference to: (i) the expression of the pro-inflammatory marker cyclooxygenase-2 (COX-2); (ii) the appearance and aggregation of Aβ 42 peptides; (iii) epigenetic mechanisms involving microRNA (miRNA) signaling that appear to be associated with disease propagation; and (iv) how direct and non-invasive analysis of the retina may help to detect and diagnose AD.

## COX-2 and brain and retinal degeneration

A family of cyclooxygenase (COX) enzymes in the brain and retina constitutes a group of prostaglandin-endoperoxide synthases (PTGSs) responsible for the formation of several prostanoid-types of pro-inflammatory mediators including prostaglandins, prostacyclins, thromboxanes, and leukotrienes (Yang and Chen, 2008; Cudaback et al., 2014). Cycooxygenase-2 (COX-2; EC 1.14.99.1; 72 kDa) is the inducible, NF-kB-regulated isotype of the PTGSs, and as the rate-limiting enzyme of the arachidonic acid cycle is up-regulated in anatomical regions of AD brain where it potentiates inflammatory neuropathology (Lukiw and Bazan, 1998; Bazan and Lukiw, 2002; Hoozemans et al., 2008; Lukiw et al., 2012a,b; Cudaback et al., 2014). COX-2 expression, mean abundance, activity, and signaling is significantly up-regulated in both AD and age-related macular degeneration (AMD), a progressive degeneration of the human retina pathologically similar in many ways to the neocortical degeneration observed in AD neocortex (Hoozemans et al., 2008; Dinet et al., 2013; Rodríguez Diez et al., 2013). Notably, subretinal injection of Aβ 42 peptides using C57BL/6J mouse models was found to significantly induce COX-2 expression up to 6-fold while compromising the integrity of the blood-brain barrier and inducing retinal inflammation, photoreceptor cell death and driving a progressive retinal degeneration (Dinet et al., 2013). While COX-2 remains a major player in the generation of oxygen radicals and lipid mediators in propagating inflammation in degenerating neocortex and retina, a third COX enzyme cyclooxygenase-3 (COX-3) may play ancillary roles in membrane-based COX signaling, however the role of COX-3 in AD and progressive neocortical and retinal disease is understudied and not well-understood (Cui et al., 2004; Wu and Wan, 2010). Importantly, pathogenic factors associated with aging or the later stages of both brain and retinal degenerative disease may be important in the course, development and progression of each disease and what cell types, tissues, or pathways may be preferentially affected (Cui et al., 2007; Cao et al., 2013; Dehabadi et al., 2014). It is interesting that as AD advances there is a progressive and sequential elevation in the pro-inflammatory gene expression marker COX-2 from the limbic system (where AD originates) into the primary visual cortex and retina, and this is accompanied by parallel elevations in Aβ 42 peptide abundance and inflammation across the entire entorhinal-primary visual cortex-thalamic-retinal axis (Cui et al., 2007; Kruck et al., 2008; Fang et al., 2009; Alexandrov et al., 2011; Cao et al., 2013; Cudaback et al., 2014). This is highly suggestive that soluble, pathogenic signaling factors such as COX-2, Aβ 42 peptides or other relatively small and mobile molecules such as miRNA (see below) may be important intercellular carriers of disease signals that eventually connect the brain and retina (Zhao et al., 2006; Tzekov and Mullan, 2014; Zhao et al., 2014).

## AD and Aβ42 peptides

All forms of AD are characterized neuropathologically by the progressive appearance of intracellular neurofibrillary tangles and dense, mostly extracellular, insoluble Aβ 42 peptide-enriched lesions known as senile plaques (Alexandrov et al., 2011; Sivak, 2013; Hardy et al., 2014). Aβ 42 peptides which form the central core of the senile plaque are generated by a complex series of secretase-mediated cleavage events from the neuronal-enriched, polytopic beta-amyloid precursor protein (β APP; Hardy et al., 2014). The very presence of Aβ 42 peptides in any tissue or physiological circuit also implicates the existence of sufficient β APP precursor and several functional membrane-associated accessory proteins—beta and gamma secretases, presenilin, and nicastrin for example—which are required for efficient Aβ 42 peptide generation (Hardy et al., 2014; Zhao et al., 2014). β APP, the enzymatic machinery required to generate Aβ 42 peptides, the Aβ 42 peptides themselves, and their fibrillar and/or oligomeric aggregates are considerably enriched in both the hippocampal CA1/association neocortex and sensory retina as AD progresses (Alexandrov et al., 2011; Ohno-Matsui, 2011; Lukiw et al., 2012a,b; Cao et al., 2013; Sivak, 2013; Zhao et al., 2014). Indeed both the progressively deposited senile plaques of AD and the drusen of AMD contain high concentrations of Aβ 42 monomers, dimers, and oligomers aggregated at their cores (Alexandrov et al., 2011; Ohno-Matsui, 2011; Lukiw et al., 2012a,b; Sivak, 2013; Zhao et al., 2014). One recent study reported Aβ 42 peptides accumulating up to 9-fold and greater over controls in the retina of advanced AD patients, and Aβ 42 peptides were found to be increased ~19-fold or greater over controls in the retinas of aging Tg2576 mice (a transgenic amyloid over-expressing murine model of AD; TgAD), and a remarkable 53-fold or more over controls in the retina of the advanced 5xFAD murine TgAD model (Lukiw et al., 2012b; Zhao et al., 2014). From these and other data it is clear that as AD or AMD initiates and progresses, Aβ 42 peptides and pro-inflammatory markers (such as COX-2) sequentially populate the hippocampus and neocortex, the primary visual cortex and then the retina. This is further supported by the temporal pattern of progressive Aβ 42 peptide deposition in TgAD models as their phenotype develops (Lukiw et al., 2012b; Tzekov and Mullan, 2014). Interestingly, the degree of visual problems reported by AD patients has a strong correlation with the degree of Aβ 42 and related amyloidogenic and pro-inflammatory markers in the primary visual cortex when short post-mortem interval brains are examined for the expression of genes involved in inflammatory neurodegeneration. The magnitude of these defects appear to mirror the temporal sequence of declining cognitive status in the AD patients, especially in late-onset AD cases with more rapid progression and in aged AD patients (Jellinger and Bancher, 1998; Thompson et al., 2003; Wilson et al., 2006; Cui et al., 2007; Tzekov and Mullan, 2014; unpublished observations). Again, these findings suggest a progressive and propagating component of AD neuropathology that extends well beyond the anatomical centers of the brain where AD originates.

## microRNA and AD propagation

One hundred and eight years of AD research has revealed that despite an entorhinal/neocortical origin for AD in the hippocampal CA1 and/or the superior temporal lobe (Brodmann area A22), AD neuropathology eventually spreads insidiously into the frontal, parietal and occipital lobes including the primary visual cortex, and then to other susceptible, anatomically-connected regions of the CNS including the retina (Alzheimer et al., 1995; Zhao et al., 2006; Cui et al., 2007; Zhao et al., 2014). The mechanism for this spreading is not well-understood, but the translocation of soluble pathogenic factors including small nucleic acids between human brain cells, and between neural cells and the cerebrospinal fluid have been reported (Zhao et al., 2006; Pogue et al., 2014). The finding that the abundance and complexity of intraneural miRNAs are often contiguous with the extracellular fluid (ECF), and that the ECF and cerebrospinal fluid (CSF) contain many of the same pathogenic miRNAs, such as the inducible, NF-kB-regulated pro-inflammatory microRNAs miRNA-9, miRNA-34a, miRNA-125b, miRNA-146a, and miRNA-155, suggests that these soluble, degeneration-associated single stranded RNAs are capable of translocating throughout the circulating fluids of the CNS and perhaps into the blood serum. It is our opinion that besides their obvious diagnostic value, pharmacological approaches aimed at down-regulating overly abundant, pathogenic miRNAs by using anti-miRNA strategies may ultimately have application in the clinical management of AD and AMD, and perhaps other diseases with an amyloidogenic and pro-inflammatory component (Cui et al., 2010; Alexandrov et al., 2011; Zhao et al., 2014).

## Concluding remarks

In summary, a picture is emerging of the evolution of the AD phenotype as an advancing, spreading or “propagating” amyloidogenic and pro-inflammatory, ultimately fatal, CNS dysfunction eventually manifesting as a more globalized disorder than previously appreciated, involving the brain's limbic system, primary visual cortex and eventually the sensory retinal systems, especially in the later stages of AD. It still remains unclear whether AD neuropathology evolves independently in the limbic system, primary visual cortex and retina, but current findings suggest that there is a “continuity” or “spreading” of the pathology from the limbic system to the entorhinal-cortex-visual cortex-retinal circuit. The cellular linkage of the hippocampal CA1 to the primary visual cortex, from the primary visual cortex to the thalamus, and from the thalamus to the retinal ganglion can involve as few as three neurons and their processes, although many more different types of neurons and complex circuitries in the retinal-primary visual cortex connectome have recently become apparent that are required for homeostatic visual processing (Cui et al., 2007; Masland, 2013; Dehabadi et al., 2014). The common neuroectodermal origins of the neocortex and retina may predispose these highly integrated, multi-neuronal layered structures to AD-type dysfunction, including the involvement of shared pathogenic pathways that drive amyloidogenesis and pro-inflammatory neurodegeneration. Importantly, the retina is the only component of the CNS that can be visualized directly and non-invasively. We propose that rigorous retinal examination including electrooculography, high resolution retinal imaging, ophthalmoscopy, optical coherence tomography (OCT) analyzing retinal nerve fiber layer (RNFL) thinning and other parameters, visual evoked potential (VEP) analysis, or combinations of these and other advanced techniques may have considerable value in the estimation and diagnosis of early AD-type change. It is our opinion that the integration of the data obtained from these multiple non-invasive techniques of the retina at various stages of AD may be of useful diagnostic value as they should be reflective of the structural and functional pathologies originating in deeper structures of the AD brain (Koch et al., 2006; Kesler et al., 2011; Vaney et al., 2012; Moreno-Ramos et al., 2013; Chang et al., 2014; Dehabadi et al., 2014; Tzekov and Mullan, 2014).

### Conflict of interest statement

The authors declare that the research was conducted in the absence of any commercial or financial relationships that could be construed as a potential conflict of interest.
